# Supplementary material for: Hot-carrier trapping preserves high quantum yields but limits optical gain in InP-based quantum dots
Source: Nat Commun. 2025 Jul 7;16:6249. doi: 10.1038/s41467-025-61511-9 (PMC12234661; doi:10.1038/s41467-025-61511-9)
Supplement: Supplementary file 1 — Supplementary Information [file 41467_2025_61511_MOESM1_ESM.pdf]

Supplementary Information for

Hot-Carrier Trapping Preserves High Quantum Yields but Limits Optical Gain in InP-Based Quantum Dots

Sander J.W. Vonk[1,2], *P. Tim Prins*[2], *Tong Wang*[3], *Jan Matthys*[4,5], *Luca Giordano*[4], *Pieter Schiettecatte*[4], *Navendu Mondal*[3], *Jaco J. Geuchies*[6,7], *Arjan J. Houtepen*[7], *Jessi E.S. van der Hoeven*[3], *Thomas R. Hopper*[3,9], *Zeger Hens*[4,5], *Pieter Geiregat*[4,5], *Artem A. Bakulin*[3], *Freddy T. Rabouw*[*1,2]

[1]Soft Condensed Matter & Biophysics, Debye Institute for Nanomaterials Science, Utrecht University, Princetonplein 1, 3584 CC Utrecht, The Netherlands

[2]Inorganic Chemistry & Catalysis, Institute for Sustainable and Circular Chemistry, Utrecht University, Universiteitsweg 99, 3584 CG Utrecht, The Netherlands

[3]Department of Chemistry and Center for Processable Electronics, Imperial College London, W120BZ London, United Kingdom

[4]Department of Chemistry, Ghent University, Krijgslaan 281, 9000 Ghent, Belgium

[5]NOLIMITS, Core Facility for Non-Linear Microscopy and Spectroscopy, Ghent University, Krijgslaan 281, 9000 Ghent, Belgium

[6]Leiden Institute of Chemistry, Leiden University, Einsteinweg 55, 2333 CC Leiden, The Netherlands

[7]Opto-Electronic Materials Section, Faculty of Applied Sciences, Delft University of Technology, van der Maasweg 9, 2629 HZ Delft, The Netherlands

[8] Materials Chemistry & Catalysis, Debye Institute for Nanomaterials Science, Utrecht University, Universiteitsweg 99, 3584 CG Utrecht, The Netherlands

[9]SLAC National Accelerator Laboratory, Menlo Park, 94043 California, United States

[*] Corresponding Author: f.t.rabouw@uu.nl

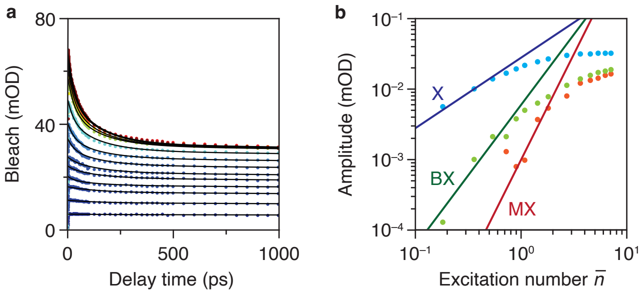

Supplementary Figure 1 | Power-dependent lifetime analysis. (a) Transient band-edge bleach traces as a function of excitation number $\overline{n} = 0$–7 (blue to red). Black lines: global fit of triexponential decay to all traces including long τ_{short}, intermediate τ_{int}, and short τ_{short} lifetime components, with varying amplitude contributions. We find $\tau_{\mathrm{int}} = 147$ ps and $\tau_{\mathrm{short}} = 29$ ps. The fitted τ_{long} is unphysically long, which we attribute to slow exciton decay (several tens of ns) on the timescales of the pump–probe experiment (< 1 ns). **(b)** Fitted amplitudes of the long (blue dots), intermediate (green dots), and long (red dots) lifetime components as a function of excitation number $\overline{n}$. The long and intermediate components follow a linear (blue line) and quadratic (green line) increase with $\overline{n}$, consistent with Poissonian absorption statistics of excitons and biexcitons, respectively. The short-lifetime component shows an even higher-order increase with $\overline{n}$ (red line shows cubic increase), indicating the formation of higher multiexcitons. Based on this we attribute the fast-decaying lifetime components to biexcitons ($\tau_{\mathrm{int}} = \tau_{\mathrm{BX}} = 147$ ps) and higher multiexcitons ($\tau_{\mathrm{short}} = \tau_{\mathrm{MX}} = 29$ ps). The biexciton lifetime is 2× longer than reported in previous studies on InP/ZnSe/ZnS QDs[S1,S2].

Supplementary Note 1 – State filling and hot-carrier losses

1.1 Absorbance bleach due to state filling in InP-based materials

In our pump–probe experiments, we observe a bleach of the ground-state absorbance A_0 due to state filling. The ground-state absorbance A_0 is given by

$$A_0 = A' g_{\mathrm{e}} g_{\mathrm{h}}, \tag{1}$$

where the product of the band-edge degeneracies of the conduction band (g_{e}) and valence band (g_{h}) gives the total number of absorption transitions, and A' is a proportionality constant containing the concentration of QDs and the oscillator strength of the transition. Upon optical excitation, band-edge states will be occupied by n_{e} electrons and n_{h} holes. Consequently, the excited-state absorbance A_* decreases to

$$A_* = A'[g_{\mathrm{e}} - \min(n_{\mathrm{e}}, g_{\mathrm{e}})][g_{\mathrm{h}} - \min(n_{\mathrm{h}}, g_{\mathrm{h}})], \tag{2}$$

as the number of absorption transitions decreases. The terms $\min(n_i, g_i)$ ensure that the excited-state absorbance does not grow negative for increasing n_i. Additionally, occupation of electrons and holes at the band edge introduce stimulated emission S_*, equivalent to negative absorbance

$$S_* = -A' \min(n_{\mathrm{e}}, g_{\mathrm{e}}) \min(n_{\mathrm{g}}, g_{\mathrm{h}}). \tag{3}$$

Here, we assumed equal oscillator strengths for stimulated emission and absorption at the probe wavelength of interest. We find the absorbance bleach normalized to the ground-state absorbance ΔA^0 as a function of n_{e} and n_{h}, given by

$$\Delta A^0(n_{\mathrm{e}}, n_{\mathrm{h}}) = \frac{A_0 - A_* - S_*}{A_0} = 1 - \frac{[g_{\mathrm{e}} - \min(n_{\mathrm{e}}, g_{\mathrm{e}})][g_{\mathrm{h}} - \min(n_{\mathrm{h}}, g_{\mathrm{h}})] - \min(n_{\mathrm{e}}, g_{\mathrm{e}}) \min(n_{\mathrm{g}}, g_{\mathrm{h}})}{g_{\mathrm{e}} g_{\mathrm{h}}}. \tag{4}$$

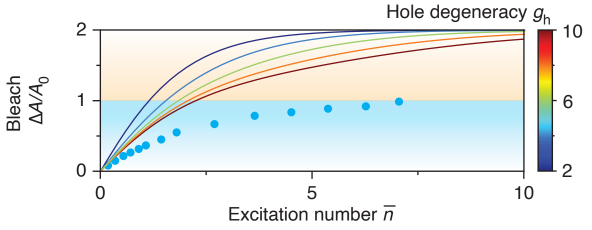

Supplementary Figure 2 | Maximum bleach for different hole degeneracies. Maximum bleach as a function of excitation number $\overline{n}$ for different hole degeneracies between $g_{\mathrm{h}} = 2$–10 (fixed electron degeneracy $g_{\mathrm{e}} = 2$). All hole degeneracies show complete population inversion at high excitation powers, showing that the experimental results on InP/ZnSe/ZnS cannot be explained by invoking a high hole degeneracy.

1.2 Conversion of photon fluence J to average number of excitations per laser pulse $\overline{n}$

We record the maximum (Figs. 2e,f) and single-exciton (Fig. 2g) absorbance bleach for varying photon fluences J to quantify the (lack of) gain in our InP-based QDs. To convert the photon fluences J to an average number of excitations per QD per laser pulse $\overline{n} = \sigma J$, we need to quantify the absorption cross section σ at the excitation wavelength.

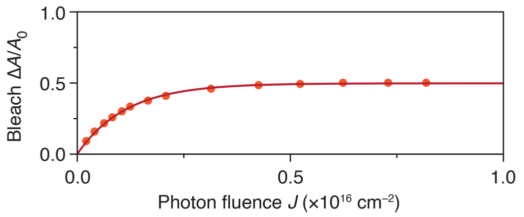

Supplementary Figure 3 | Saturation of the single-exciton bleach. Single-exciton bleach (red dots) of our InP/ZnSe/ZnS QD sample as a function of photon fluence J using 515-nm excitation. We fit the absorption cross section $\sigma_{\mathrm{X}} = 0.80\times10^{-15}$ cm^2 from the saturation of the single-exciton bleach (red solid line, equation 5).

A common procedure to retrieve the absorption cross section, is through a saturation measurement of the single-exciton bleach ΔA_{X} (red dots) as a function of photon fluence J. Suppl. Fig. 3 shows the single-exciton bleach (red dots, normalized to A_0) as a function of the photon fluence J for 515-nm excitation. Theoretically, the single-exciton bleach follows from the probability of forming at least one exciton per laser pulse $P_{\geq 1} = 1 - p(0, \overline{n})$, and the bleach due to a single excitation $\Delta A^0(1, 1)$:

$$\Delta A_{\mathrm{X}} = \Delta A^0(1, 1)[1 - p(0, \sigma J)], \tag{5}$$

which we fit to the experimental data, using $\Delta A^0(1, 1)$ and $\sigma = \sigma_{\mathrm{X}}$ as the fit parameters (solid red line). We find $\sigma_{\mathrm{X}} = 0.80 \times 10^{-15}$ cm^2. Later in this Supplementary Note, we show that hot-carrier trapping introduces cascades of Auger processes, which lowers the saturation value of the single-exciton bleach compared to what we would expect from a QD without losses (Supplementary Figs. S3b–d). This might introduce a systematic error in the fitted absorption cross section σ_{X}, which depends on the exact value of the hot-carrier trapping probability P_{trap}.

To circumvent this issue, we calculate the absorption cross section through Maxwell–Garnett effective-medium theory for a core–shell QD geometry as reported in Ref. S3. We calculate the absorption cross section σ for our QDs at 350 nm where size-quantization effects are negligible. The analytical expressions presented in Ref. S3 hold for spherical core–shell particles. We model ours as particles with a core of InP[S4] and a shell with the permittivity of ZnSe[S5], surrounded by toluene[S6]. All permittivity values are

taken at 350 nm. The outer ZnS shell is neglected, because the real part of the refractive index of ZnS is similar to ZnSe at 350 nm, while ZnS has negligible absorption. We set the total QD volume equal to that of a tetrahedron with an edge length of $L = 10.2$ nm, consistent with Fig. 1a, and the core radius to 1.5 nm. Finally, we find the absorption-cross section at 515 nm $\sigma_{\mathrm{MG}} = 0.92 \times 10^{-15}$ cm^2, using the calculated absorption cross section at 350 nm and the relative absorbance from the linear absorbance spectrum (Fig. 1b). We observe that the calculated absorption cross section σ_{MG} is comparable to the one obtained from the single-exciton bleach saturation σ_{X}.

The qualitative observation that hot-carrier losses occur in our InP/ZnSe/ZnS QD sample, does not depend on the exact value of the absorption cross section. This can be observed in Fig. 1e of the main text, where two black lines for the state-filling model without losses are plotted using σ_{X} and σ_{MG}, respectively. Therefore, in all other Figures of the main text and Supplementary Information, we simply estimate $\overline{n}$ from the mean absorption cross section σ, *i.e.* $\sigma = (\sigma_{\mathrm{X}} + \sigma_{\mathrm{MG}})/2$, obtained through these two separate approaches.

1.3 Ideal case: no losses in the cooling phase

First, we consider the ideal scenario in which all hot charge carriers cool down to the band edge. Hence, n excitations generated by the laser result in a "maximum bleach" $\Delta A^0_{\mathrm{max}}$, at the end of the cooling phase, due to $n_{\mathrm{e}} = n$ electrons and $n_{\mathrm{h}} = n$ holes. We calculate this by averaging over the Poisson distribution $p(n, \overline{n})$ with mean $\overline{n}$:

$$\Delta A^0_{\mathrm{max}}(\overline{n}) = \sum_{n=0}^{\infty} p(n, \overline{n}) \Delta A^0(n, n) \tag{6}$$

We plot equation 6 for $g_{\mathrm{e}} = 2$ and $g_{\mathrm{h}} = 4$ in Fig. 1e of the main text. Similarly, we find the single-exciton bleach

$$\Delta A^0_{\mathrm{X}}(\overline{n}) = \Delta A^0(1, 1)[1 - p(0, \overline{n})], \tag{7}$$

from the probability that at least 1 excitation was formed $p_{\geq 1} = [1 - p(0, \overline{n})]$ and the bleach of a single excitation at the band edge $\Delta A^0(1, 1)$.

1.4 Hot-carrier trapping and trap-assisted Auger quenching

The experiments are inconsistent with the model of the previous section and show evidence for losses during the cooling phase (Fig. 1e). Including a simple constant hot-carrier-trapping probability is not enough to match the experiment, because the losses increase with increasing $\overline{n}$. To reproduce the saturation of the maximum bleach in our experiment (Figs. 1e,f), we propose a positive-feedback mechanism. In this mechanism, trapping of a charge carrier introduces a very efficient nonradiative recombination pathway to the *other* excitations in the QD. More specifically, we assume that the trapped carrier is a very efficient Auger acceptor that quenches all other excitations in the QD within the timescale of cooling (picosecond). For a highly excited QD, this requires a cascade of Auger processes. At the end of a cooling phase following this scenario (scenario B, Suppl. Fig. 4a), only the single charge-separated excitation remains. Alternatively (scenario A, Suppl. Fig. 4a) all charge carriers may cool down to the band edge successfully without trapping, in which case no trap-assisted Auger recombination occurs.

In this picture, starting with n excitations, we either end up in scenario A (Suppl. Fig. 4a) where all n excitations successfully cooled down, or in scenario B (Suppl. Fig. 4a) where only one charge-separated excited state remains. The probability P_{A} of ending in scenario A is

$$P_{\mathrm{A}} = (1 - P_{\mathrm{trap}})^n, \tag{8}$$

which decreases with the trapping probability P_{trap} and the number of excitations n. Conversely, the probability of ending up in scenario B is

$$P_{\mathrm{B}} = 1 - P_{\mathrm{A}}. \tag{9}$$

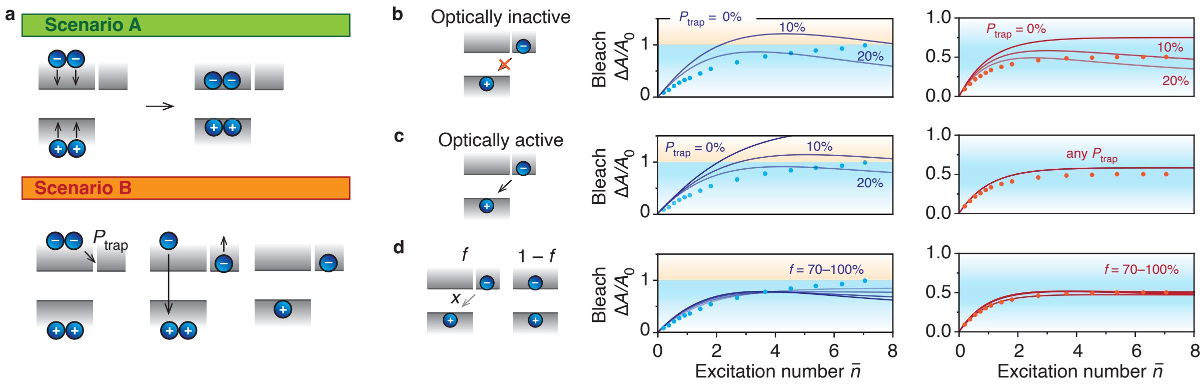

Supplementary Figure 4 | Hot-carrier trapping leading to losses in InP-based QDs. (a) Proposed model for cooling in InP-based QDs including hot-carrier trapping. Successful cooling of all n excitations (scenario A) results in n excitations at the band-edge. However, trapping of a single carrier with probability P_{trap} quenches all remaining excitations (scenario B), leading to a single charge-separated excitation after the cooling phase. **(b)** Maximum bleach (middle) and single-exciton bleach (right) for varying trapping probability P_{trap} between 0 and 20%, assuming an optically inactive trap state (left). These plots are identical to those in Fig. 1e,g of the main text. We observe that the experimental maximum bleach and single-exciton bleach can both be matched using a trapping probability of $P_{\text{trap}} = 10$–15%. **(c)** Same as **b**, but including the observation (Figs. 3,4 of the main text) that the charge-separated exciton is optically active. Here, we set the oscillator strength equal to a band-edge exciton. This effectively increases the degeneracy of the band-edge state by one, which decreases the initial slope of the maximum bleach and single-exciton bleach as a function of $\overline{n}$. For an optically active trap state, the charge-separated exciton and the regular exciton contribute equally to the bleach, making the single-exciton bleach independent of the trapping probability P_{trap}. **(d)** Same as **b** and **c**, but now including the observation (Fig. 4) that only a fraction f of the QDs exhibits hot-carrier trapping at any particular moment, while the other fraction $(1-f)$ has no hot-carrier losses. Here, we find a good match between the model and experimental data by fitting the relative oscillator strength x and trapping probability P_{trap} while fixing f at 70, 80, 90, or 100%. From the fit procedure, we find average trapping probabilities of $\overline{P}_{\text{trap}} = fP_{\text{trap}} = 26$–50% and relative oscillator strengths of $x = 20$–50%, consistent with our experimental findings in Figs. 2, 3, and 4 of the main text.

1.4.1 Model 1: Trapping into an optically inactive trap state

The expected bleach following hot-carrier trapping depends on the optical properties of the trap state. In Fig. 1f,g of the main text and Suppl. Fig. 4b, we make the simplest possible assumption: the trap state is optically inactive. The trapped charge carrier does not contribute to absorption or stimulated emission.

The absorbance bleach in scenario A of section (ΔA_{A}) is simply equal to ΔA^0 obtained without losses, given in equation 4. In scenario B, however, the absorbance bleach of the charge-separated excitation

$$\Delta A_{\text{B}} = \Delta A^0(0,1) = 1 - \frac{g_{\text{e}}(g_{\text{h}}-1)}{g_{\text{e}}g_{\text{h}}}, \tag{10}$$

is independent of n, because only the band-edge hole bleaches the absorbance. Equivalently to the procedure in equation 6, we average over a Poisson distribution to find the absorbance bleach as a function of $\overline{n}$. In our model with trap-assisted Auger quenching, the probabilities of scenarios A and B (P_{A} and P_{B}) depend on n and the trapping probability P_{trap}. We use these probabilities to find the maximum bleach and single-exciton bleach as a function of $\overline{n}$:

$$\Delta A_{\max}(\overline{n}, P_{\text{trap}}) = \sum_{n=1}^{\infty} p(n,\overline{n})[\Delta A_{\text{A}}(n,n)P_{\text{A}}(n,P_{\text{trap}}) + \Delta A_{\text{B}}P_{\text{B}}(n,P_{\text{trap}})] \tag{11}$$

$$\Delta A_{\rm X}(\overline{n}, P_{\rm trap}) = \sum_{n=1}^{\infty} p(n,\overline{n})[\Delta A_{\rm A}(1,1)P_{\rm A}(n, P_{\rm trap}) + \Delta A_{\rm B} P_{\rm B}(n, P_{\rm trap})]. \tag{12}$$

We can simplify the expressions by using $\sum_{n=1}^{\infty} p(n,\overline{n})P_{\rm B}(n, P_{\rm trap}) = 1 - {\rm e}^{-\overline{n}P_{\rm trap}}$ to obtain

$$\Delta A_{\rm max}(\overline{n}, P_{\rm trap}) = \Delta A_{\rm B}\left(1 - {\rm e}^{-\overline{n}P_{\rm trap}}\right) + \sum_{n=1}^{\infty} p(n,\overline{n})\Delta A_{\rm A}(n,n)P_{\rm A}(n, P_{\rm trap}) \tag{13}$$

$$\Delta A_{\rm X}(\overline{n}, P_{\rm trap}) = \Delta A_{\rm B}\left(1 - {\rm e}^{-\overline{n}P_{\rm trap}}\right) + \sum_{n=1}^{\infty} p(n,\overline{n})\Delta A_{\rm A}(1,1)P_{\rm A}(n, P_{\rm trap}). \tag{14}$$

We plot eqs. 13,14 in Suppl. Fig. 4b and find that the trap-assisted Auger model matches both the experimental maximum bleach and single-exciton bleach reasonably well with a trapping probability $P_{\rm trap} \approx 0.15$.

1.4.2 Model 2: Trapping into an optically active trap state

In Figs. 3,4 of the main text, we found signatures of trap-state emission on the ensemble and single-QD level. These findings show that the trap state can be optically active. We include this in our model using a parameter $x \in [0,1]$ describing the relative oscillator strength of the trap-to-valence-band transition compared to the band-edge transitions. This changes the absorbance bleach due to scenario A to

$$\Delta A_{\rm A}(n_{\rm e}, n_{\rm h}, x) = 1 - \frac{[g_{\rm e} - \min(n_{\rm e}, g_{\rm e})][g_{\rm h} - \min(n_{\rm h}, g_{\rm h})] - \min(n_{\rm e}, g_{\rm e})\min(n_{\rm h}, g_{\rm h}) + x[g_{\rm h} - \min(n_{\rm h}, g_{\rm h})]}{g_{\rm e}g_{\rm h} + xg_{\rm h}}, \tag{15}$$

because now the trap state can contribute to absorption. Similarly, we find that the absorbance bleach of the charge-separated excitation in scenario B changes to

$$\Delta A_{\rm B}(x) = 1 - \frac{g_{\rm e}(g_{\rm h} - 1) - x}{g_{\rm e}g_{\rm h} + xg_{\rm h}}, \tag{16}$$

where now the charge-separated excitation contributes to stimulated emission depending on the oscillator strength of the transition x. We obtain the maximum bleach and single-exciton bleach by averaging over the Poisson distribution:

$$\Delta A_{\rm max}(\overline{n}, P_{\rm trap}, x) = \Delta A_{\rm B}(x)\left(1 - {\rm e}^{-\overline{n}P_{\rm trap}}\right) + \sum_{n=1}^{\infty} p(n,\overline{n})\Delta A_{\rm A}(n,n,x)P_{\rm A}(n, P_{\rm trap}) \tag{17}$$

$$\Delta A_{\rm X}(\overline{n}, P_{\rm trap}, x) = \Delta A_{\rm B}(x)\left(1 - {\rm e}^{-\overline{n}P_{\rm trap}}\right) + \sum_{n=1}^{\infty} p(n,\overline{n})\Delta A_{\rm A}(1,1,x)P_{\rm A}(n, P_{\rm trap}), \tag{18}$$

Suppl. Fig. 4c shows the experimental maximum bleach (blue dots) and single-exciton bleach (red dots) and the model of hot-carrier trapping with trap-assisted Auger quenching for trapping probabilities $P_{\rm trap} = 0,\ 10,\ 20\%$ and trap-state oscillator strength $x = 1$. Interestingly, we observe that the single-exciton bleach is independent of the trapping probability, because the charge-separated excitation $\Delta A_{\rm B}(1)$ and the single delocalized excitation $\Delta A_{\rm A}(1,1,1)$ bleach the absorbance equally. The experiments in Figs. 3,4 show that in our InP-based sample the oscillator strength varies in time and over the ensemble. The average x will be somewhere between 0 and 1. We observe that the models show a reasonable agreement with the experiment, irrespective of whether the trap state is inactive ($x = 0$, Suppl. Fig. 4b) or active (Suppl. Fig. 4c).

1.4.3 Model 3: Fraction of QDs with (partially) optically active trap state

In general, colloidal dispersions of QDs show particle-to-particle variations on the single-QD level. For example, earlier experimental work on Cd-based samples found a "dark fraction", a subpopulation of QDs with zero emission efficiency. Inspired by this experimental finding, we investigate whether we can better match the experimental bleach data by assuming subpopulations in our sample with a different trapping probability P_{trap}. Going for the simplest possible implementation of this idea, we assume that a fraction f of QDs shows hot-carrier trapping with probability P_{trap} while the remaining fraction $1-f$ has no hot-carrier trapping (*i.e.* $P_{\mathrm{trap}}=0$). In this case, the maximum bleach and single-exciton bleach are given by

$$\Delta A_{\max}(\overline{n}, P_{\mathrm{trap}}, x, f) = f\Delta A^{(2)}_{\max}(\overline{n}, P_{\mathrm{trap}}, x) + (1-f)\Delta A^{0}_{\max} \tag{19}$$

$$\Delta A_{\mathrm{X}}(\overline{n}, P_{\mathrm{trap}}, x, f) = f\Delta A^{(2)}_{\mathrm{X}}(\overline{n}, P_{\mathrm{trap}}, x) + (1-f)\Delta A^{0}_{\mathrm{X}}, \tag{20}$$

$\Delta A^{(2)}_{\max}(\overline{n}, P_{\mathrm{trap}}, x)$ and $\Delta A^{(2)}_{\mathrm{X}}(\overline{n}, P_{\mathrm{trap}}, x)$ denote the expressions we derived above in model 2.

In Suppl. Fig. 4d, we vary the fraction f between 70–100% and fit the remaining parameters (relative oscillator strength x and trapping probability P_{trap}) to find the optimal match with the data. The match with the experiment is improved (compare to Suppl. Fig. 4c) assuming two sub-populations. The best agreement is found for average trapping probabilities of $\overline{P}_{\mathrm{trap}} = fP_{\mathrm{trap}} = 26$–$50\%$ and relative oscillator strengths of $x = 0.2$–0.5. The match with the experiment might be improved further by including a more advanced model for the distribution of trapping probabilities, but without further experimental data it is impossible to justify the choice for any particular model.

1.5 Gain spectrum following model 3

In our excited-state absorbance spectra after cooling (Fig. 1d), a modest gain feature develops redshifted from the linear absorption spectrum due to a Stokes shift between absorption and stimulated emission.[S2] Here, we implement a Stokes shift ΔE between absorption and stimulated emission into our state-filling model including hot-carrier trapping. The energy-dependent contributions of absorption and stimulated emission for scenarios A and B become:

$$A_{\mathrm{A}}(E, n_{\mathrm{e}}, n_{\mathrm{h}}, x) = \tilde{A}(E)\frac{[g_{\mathrm{e}} - \min(n_{\mathrm{e}}, g_{\mathrm{e}})][g_{\mathrm{h}} - \min(n_{\mathrm{h}}, g_{\mathrm{h}})] + x[g_{\mathrm{h}} - \min(n_{\mathrm{h}}, g_{\mathrm{h}})]}{g_{\mathrm{e}}g_{\mathrm{h}} + xg_{\mathrm{h}}} \tag{21}$$

$$S_{\mathrm{A}}(E, \Delta E, n_{\mathrm{e}}, n_{\mathrm{h}}, x) = -\tilde{S}(E, \Delta E)\frac{\min(n_{\mathrm{e}}, g_{\mathrm{e}})\min(n_{\mathrm{h}}, g_{\mathrm{h}})}{g_{\mathrm{e}}g_{\mathrm{h}} + xg_{\mathrm{h}}} \tag{22}$$

$$A_{\mathrm{B}}(E, x) = \tilde{A}(E)\frac{g_{\mathrm{e}}(g_{\mathrm{h}} - 1)}{g_{\mathrm{e}}g_{\mathrm{h}} + xg_{\mathrm{h}}} \tag{23}$$

$$S_{\mathrm{B}}(E, \Delta E, x) = -\tilde{S}(E, \Delta E)\frac{x}{g_{\mathrm{e}}g_{\mathrm{h}} + xg_{\mathrm{h}}} \tag{24}$$

where $\tilde{A}(E)$ is a Gaussian function fitted to the linear absorption spectrum, and $\tilde{S}(E, \Delta E)$ is the same spectrum but Stokes shifted by ΔE. In the fraction of QDs with an active trap, the excited-state absorbance directly after the cooling phase is given by:

$$A_{\max}(E, \Delta E, \overline{n}, x) = \sum_{n=0}^{\infty} p(n, \overline{n})\{P_{\mathrm{A}}[A_{\mathrm{A}}(E, n, n, x) + S_{\mathrm{A}}(E, \Delta E, n, n, x)] + P_{\mathrm{B}}[A_{\mathrm{B}}(E, x) + S_{\mathrm{B}}(E, \Delta E, x)]\}. \tag{25}$$

Similarly, the absorbance, stimulated emission, and total absorbance directly after the cooling phase and without hot-carrier trapping (model 0) is given by:

$$A^0(E, n_\mathrm{e}, n_\mathrm{h}) = \tilde{A}(E)\frac{[g_\mathrm{e} - \min(n_\mathrm{e}, g_\mathrm{e})][g_\mathrm{h} - \min(n_\mathrm{h}, g_\mathrm{h})]}{g_\mathrm{e} g_\mathrm{h}} \quad (26)$$

$$S^0(E, \Delta E, n_\mathrm{e}, n_\mathrm{h}) = -\tilde{S}(E, \Delta E)\frac{\min(n_\mathrm{e}, g_\mathrm{e})\min(n_\mathrm{h}, g_\mathrm{h})}{g_\mathrm{e} g_\mathrm{h}} \quad (27)$$

$$A^0_\mathrm{max}(E, \Delta E, \overline{n}) = \sum_{n=0}^{\infty} p(n, \overline{n})\{[A^0(E, n, n) + S^0(E, \Delta E, n, n)]\}. \quad (28)$$

We find the total excited-state absorbance by adding the contributions of the fraction f QDs with hot-carrier trapping and the fraction $1 - f$ without trapping:

$$A_\mathrm{tot,\ max}(E, \Delta E, \overline{n}, x, f) = f A_\mathrm{max}(E, \Delta E, \overline{n}, x) + (1 - f)A^0_\mathrm{max}(E, \Delta E, \overline{n}). \quad (29)$$

In Fig. 5 of the main text we use equation 29 to match the excited-state absorbance spectra obtained in the experiment.

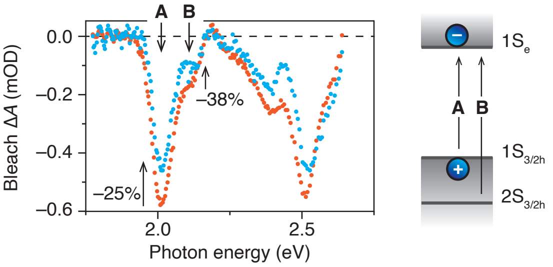

Supplementary Figure 5 | Benchmark pump–push–probe experiments on CdSe/CdS/ZnS QDs. Absorbance bleach spectrum ($E_\mathrm{pump} = 3.1$ eV and $\overline{n} = 0.1$) of benchmark CdSe/CdS/ZnS QDs before (red dots) and after a 0.95 eV push pulse at maximum bleach loss (blue dots). The marked bleach features are assigned to the **A** $1\mathrm{S_e}1\mathrm{S}_{3/2,\mathrm{h}}$ and **B** $1\mathrm{S_e}2\mathrm{S}_{3/2,\mathrm{h}}$ absorption transitions, which have a shared electron energy level but different hole levels. The loss of absorbance bleach of transition **B** shows that the electron is promoted to a higher energy level by the push pulse since excited state absorption of the hole would leave the absorbance bleach of this transition unaffected. The relative absorbance bleach loss $\Delta\Delta A/\Delta A$ for transition **A** (−25%) and **B** (−38%) are consistent with excited-state absorption of the electron, given the degeneracies of the electron and hole levels ($g_{1\mathrm{S_e}} = 2$ and $g_{1\mathrm{S_h}} = g_{2\mathrm{S_h}} = 4$).

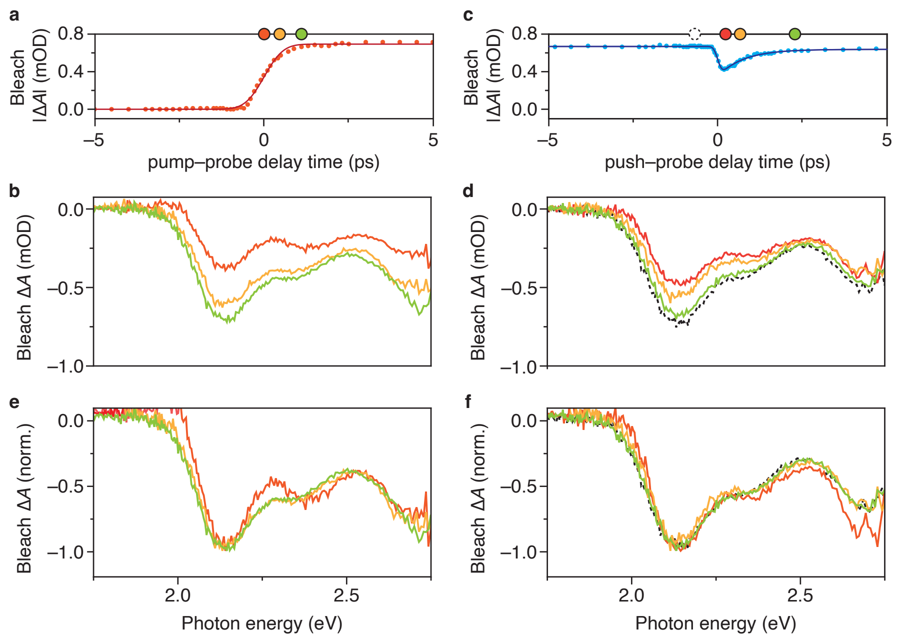

Supplementary Figure 6 | Bleach spectrum during cooling after pump or push. (a) The absorbance bleach averaged over 565–600 nm as a function of delay time with respect to pump excitation at 3.1 eV. The ingrowth reveals carrier cooling. **(b)** Same, but with respect to the push excitation at 0.52 eV. **(c)** Bleach spectra for three selected delay times following the pump pulse, as indicated in panel a. **(d)** Same, but following the push pulse. The dashed line is the bleach spectrum before the push. We observe that the bleach grows in more or less evenly over the full spectral range in both panels **c** and **d**. In panel **d**, the bleach does not recover completely, which we ascribe to hot-electron losses. **(e,f)** Normalized bleach spectra at the same delay times as in panels **c,d**, respectively. These show that the bleach grows in evenly over the spectral range between 2.0 eV and 2.5 eV. Following the assignments of Ref. S7, we assign the bleach peak at 2.15 eV to the $1S_h$–$1S_e$ transition and the one at 2.4 eV to the $2S_h$–$1S_e$ transition. Their even recovery is thus consistent with excitation of the electron, which affects the two transitions equally. We do not observe transient bleach signatures at higher energy due to filling of the $1P_e$ electron level. This unexpected absence of clear $1P_e$ signals indicate that the $1P_e$ energy falls within the ZnSe shell conduction band.[S7] Hence, the high degeneracy of shell-delocalized states and reduced overlap with hole states reduces the contribution of the $1P_e$ level to (transient) absorption spectra. The overlap of different valence-band levels with the $1S_e$ level, on the other hand, is good. This, combined with the significant overall broadening of spectral features of InP QDs, explain why bleach spectra change evenly over a broad spectral range during cooling and no clear $1P_e$ signals are observed. The similarity of the spectra following pump (panel **e**) and push (panel **f**) excitation indicate that, in both experiments, the cooling dynamics are limited by electron cooling over the relatively large $1P_e$–$1S_e$ gap as the slowest process, with a time constant of a few hundred fs.[S8,S9]

Supplementary Note 2 – Hot-carrier losses from pump–push–probe experiments

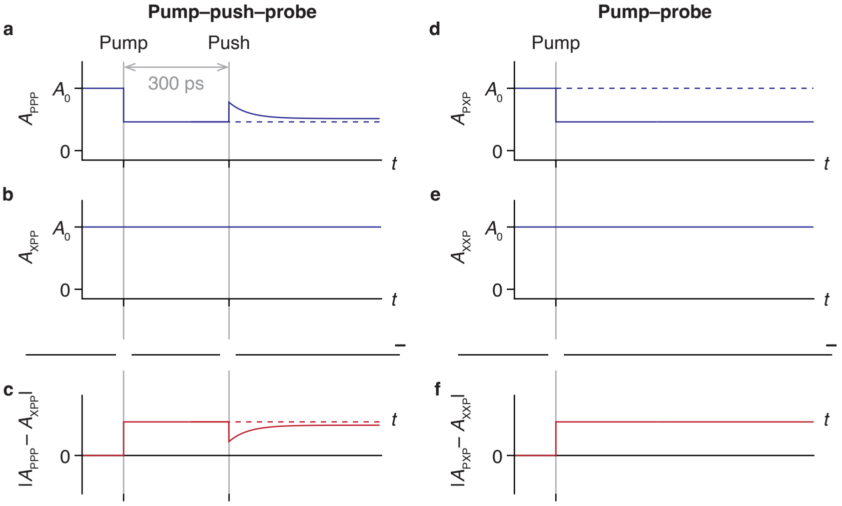

Supplementary Figure 7 | Data-recording procedure for pump–push–probe and pump–probe transients. (a)–(c) Schematic of the data-recording procedure in pump–push–probe experiments. Here, and in what follows, we use a X to indicate blocked pump (XPP), push (PXP), probe (PPX), or any other combination. By placing a chopper in the optical path of the pump laser, we directly measure the differential absorbance (**c**, $A_{\rm PPP} - A_{\rm XPP}$) between pump on (**a**, $A_{\rm PPP}$) and pump off (**b**, $A_{\rm XPP}$). In $A_{\rm PPP}$ **a**, excitations formed by the pump decrease the absorbance (ground-state absorbance A_0 as reference). 300 ps later, the push instantaneously recovers part of the absorbance by pushing the electron to a hot-carrier state. Because of hot-carrier trapping, part of the initial absorbance bleach is lost after cooling and the absorbance does not reach the absorbance before the push (dashed line). Without the pump $A_{\rm XPP}$ **b**, the push does (to first order) not interact with the sample and the absorbance is unaffected. The traces in Fig. 2 of the main text are normalised differential absorbance traces such as those in panel **c** and Suppl. Fig. 8c. **(d)–(f)** Same as **a–c**, but for the pump–probe experiments.

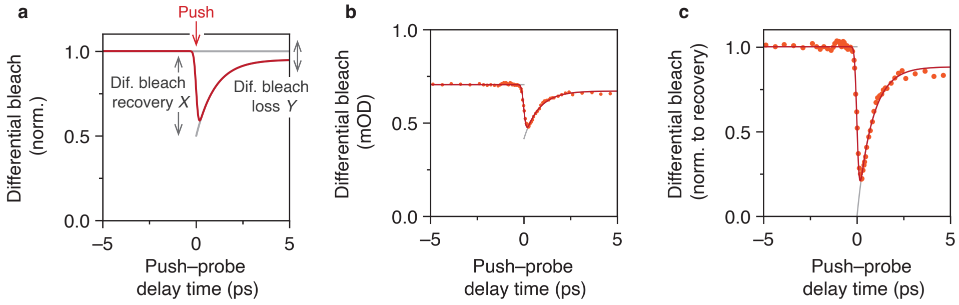

Supplementary Figure 8 | Characterizing hot-carrier losses from PPP experiments. (a) Schematic transient of the band-edge absorbance (equation 30, gray line) in a pump–push–probe (PPP) experiment. A fraction X of the bleach recovers due to the push pulse while only a fraction Y returns over the following few picoseconds. In a real experiment, the bleach recovery, hot-carrier losses, and cooling occur on timescales similar to the instrument-response function. To extract the bleach recovery X and bleach return Y from an experiment we convolve equation 30 with the instrument-response (equation 31, red line). **(b)** Experimental transient of the band-edge absorbance in a PPP experiment (red dots) of InP/ZnSe/ZnS QDs. We extract the bleach recovery and return by fitting equation 31 using an instrument-response width of 100 fs. **(c)** Same as **b**, but normalized to the bleach recovery X to visualize the hot-carrier losses.

We characterise hot-carrier losses using pump–push–probe experiments. In these experiments, we measure the differential absorbance $A_{\mathrm{PPP}} - A_{\mathrm{XPP}}$ at variable delay times t between the push pulse and the probe (Suppl. Fig. 8a). Such a transient (gray line) is given by

$$|\Delta A(t)| = \Delta A^0 \exp(-k_{\mathrm{R}}t)\{1 - XH(t) + (X - Y)H(t)[1 - \exp(-k_{\mathrm{C}}t)]\}, \tag{30}$$

where $H(t)$ is the Heaviside step function, ΔA^0 is the absorbance bleach generated by the pump pulse that is still left after the 300-ps waiting time, X is the fractional recovery by excited-state absorption of the electron, Y is the fractional loss after cooling with rate constant k_{C}, and k_{R} is the exciton recombination rate. Typically, the exciton recombination rate is much slower than cooling. InP-based QDs are special compared to other materials (Figs. 2b–d) because the differential-bleach does not return completely due to hot-carrier losses, *i.e.* $Y < X$. In a real experiment, the recovery, hot-carrier losses, and cooling all happen on timescales similar to the instrument-response function of the experimental setup. To extract the differential-bleach recovery X and loss Y from an experiment, we convolve equation 30 with a Gaussian instrument-response function and find

$$\begin{aligned}|\Delta A(t)| = \Delta A^0 \exp(-k_{\mathrm{R}}t)\Big(1 - \frac{X}{2}\Big[1 + \mathrm{erf}\Big(\frac{t}{\sqrt{2}\delta}\Big)\Big] + \\ \frac{X - Y}{2}\Big\{1 + \mathrm{erf}\Big(\frac{t}{\sqrt{2}\delta}\Big) - \exp\Big[\frac{k_{\mathrm{C}}}{2}(k\delta^2 - 2t)\Big]\Big[1 - \mathrm{erf}\Big(\frac{k_{\mathrm{C}}\delta^2 - t}{\sqrt{2}\delta}\Big)\Big]\Big\}\Big),\end{aligned} \tag{31}$$

where δ is the width of the instrument-response function, and $\mathrm{erf}(x)$ is the error function. We fit equation 31, using $\delta = 100$ fs, to the experimental data (Suppl. Fig. 8b) to extract the fractional recovery $X = 0.38$ and the loss $Y = 0.03$. We find that $Y/X = 9\%$ of the hot carriers are lost during cooling. Note that neglecting the instrument-response function in the fitting procedure would underestimate the differential-bleach recovery X (compare gray and red solid lines in Suppl. Fig. 8b). In the main text, we normalize the transients to the recovery (Suppl. Fig. 8c and Fig. 2) to easily visualize the hot-carrier losses.

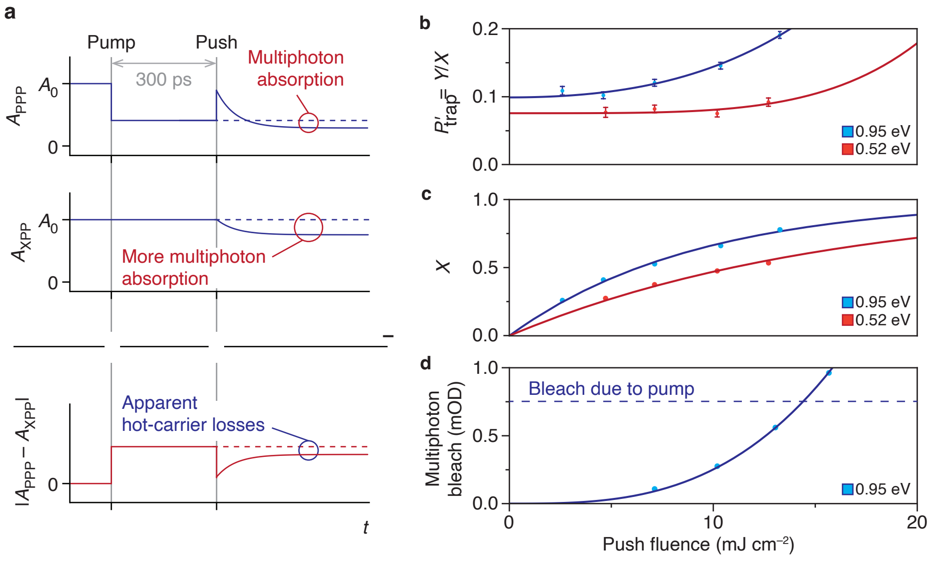

Supplementary Figure 9 | Hot-carrier losses and multiphoton absorption in pump–push–probe experiments. (a) Multiphoton absorption can look like hot-carrier losses in PPP experiments. In the differential absorbance scheme—assuming *no* hot-carrier trapping—XPP (middle) induces more absorbance bleach by multiphoton absorption compared to PPP (top), as more QDs are in the ground state upon arrival of the push pulse. This leads to apparent hot-carrier losses in the differential absorbance $A_{\mathrm{PPP}} - A_{\mathrm{XPP}}$ because of a difference in magnitude of multiphoton absorption. **(b)** Apparent hot-carrier trapping probability P'_{trap} as a function of push fluence for 0.95 eV (blue) and 0.52 eV (red) push energies. P'_{trap} is independent of push power for small push fluences for both push energies. Solid lines: fits to the data using equation 36, the recovery X from panel **c**, and $n_{\mathrm{low}} = 5$ and $n_{\mathrm{high}} = 3$ for order of the multiphoton absorption. The fits reproduce the nonlinear increase of P'_{trap} with push fluence, showing that multiphoton absorption directly influences the measurement at higher push fluences. Error bars represent one standard error and are propagated from the fit to the cooling dynamics. **(c)** Fractional bleach loss X as a function of push fluence J. Solid line: fits to data with $X(J) = 1 - \exp(-aJ)$, to account for the saturation of the push-induced bleach loss at $X = 1$. **(d)** Absorbance bleach in a control push–probe experiment with a sub-bandgap push pulse ($E_{\mathrm{push}} = 0.95$ eV) and no prior pump pulse on InP/ZnSe/ZnS QDs for varying push fluences. Dashed blue line: absorbance bleach due to the 3.1-eV pump pulse at the fluence used in the PPP experiments of the main text. The absorbance bleach shows a cubic dependence on push fluence (blue solid line) showing that 3-photon absorption populates the band-edge.

Pushing excited charge carriers to hot-carrier states requires high push fluences, which potentially introduces additional band-edge excitations by multiphoton absorption. As such, the differential-bleach recovery X and loss Y may be affected, introducing systematic errors in the apparent hot-carrier trapping probability P'_{trap}. Here, we will discuss the implications of multiphoton absorption in pump–push–probe measurements and how its influence can be minimized and quantified.

In what follows, we use a three-letter notation to indicate a blocked pump (XPP), push (PXP), probe (PPX), or any other combination of light pulses. In our experiments, we measure the differential absorbance, $A_{\mathrm{PPP}} - A_{\mathrm{XPP}}$, using a chopper to correct for push-induced optical artefacts such as the Stark effect (Suppl. Fig. 10). Interband multiphoton absorption can introduce apparent hot-carrier losses, as is schematically illustrated in Suppl. Fig. 9a. Here, XPP induces more absorbance bleach by multiphoton absorption of the push pulse compared to PPP, as more QDs are in the ground state upon arrival of the push pulse. Indeed, this leads to apparent hot-carrier losses in the differential bleach $A_{\mathrm{PPP}} - A_{\mathrm{XPP}}$—even if all hot carriers cool down with unity efficiency—simply because of a difference in interband multiphoton absorption.

Next, we will derive analytical expressions for the influence of both hot-carrier trapping and multiphoton absorption on the differential absorbance $A_{\mathrm{PPP}} - A_{\mathrm{XPP}}$. The PPP and XPP absorbance bleach

directly after arrival of the push pulse ($t = 0$; superscript 0) are given by

$$\Delta A^0_{\mathrm{PPP}} \propto P_{\mathrm{pump}}(1 - X) \quad (32)$$

$$\Delta A^0_{\mathrm{XPP}} = 0 \quad (33)$$

where P_{pump} is the probability that the pump pulse forms an excitation, and X is the fractional bleach loss, which increases linearly (until saturation; Suppl. Fig. 9c) with push fluence J. Multiphoton absorption does not contribute to the PPP or XPP bleach at $t = 0$ as the excitations need to cool down before they influence the band-edge absorbance. After cooling (late time; superscript ∞), the PPP and XPP bleach contain contributions of hot-carrier trapping and multiphoton absorption:

$$\Delta A^{\infty}_{\mathrm{PPP}} \propto P_{\mathrm{pump}}(1 - P_{\mathrm{trap}}X) + (1 - P_{\mathrm{pump}})P_{\mathrm{push}} + P_{\mathrm{pump}}P'_{\mathrm{push}} \quad (34)$$

$$\Delta A^{\infty}_{\mathrm{XPP}} \propto P_{\mathrm{push}} \quad (35)$$

where a fraction P_{trap} of X is lost because of hot-carrier trapping, P_{push} is the probability of multiphoton excitation by the push pulse of a QD in the ground state, and P'_{push} the probability of multiphoton excitation by the push pulse of a QD already in the excited state. Both P_{push} and P'_{push} increase superlinearly with push fluence J^n, with n (≥ 3 in our experiments) the order of the absorption process. From this, we can compute the apparent trapping probability P'_{trap}

$$P'_{\mathrm{trap}} = \frac{Y}{X} = \frac{\Delta A_{\mathrm{PP}} - (\Delta A^{\infty}_{\mathrm{PPP}} - \Delta A^{\infty}_{\mathrm{XPP}})}{\Delta A_{\mathrm{PP}} - (\Delta A^{0}_{\mathrm{PPP}} - \Delta A^{0}_{\mathrm{XPP}})} = P_{\mathrm{trap}} + \frac{P_{\mathrm{push}} - P'_{\mathrm{push}}}{X} \quad (36)$$

where $\Delta A_{\mathrm{PP}} = A_{\mathrm{PXP}} - A_{\mathrm{XXP}} = P_{\mathrm{pump}}$. The resulting expression consists of the fluence-independent real trapping probability P_{trap} and a fluence-dependent term, *i.e.* $P'_{\mathrm{trap}} = P_{\mathrm{trap}} + AJ^{n-1}$, due to multiphoton absorption. This expression shows that the effect of multiphoton absorption can be minimized by minimizing the push fluence J. We experimentally validate equation 36 by performing push-fluence-dependent PPP measurements. Suppl. Fig. 9b shows the apparent trapping probability P'_{trap} as a function of push fluence for low (red, 0.52 eV) and high (blue, 0.95 eV) push energies. For both push energies we observe a constant P'_{trap} at low push fluences and a nonlinear increase at higher push fluences. Next, we determine the fluence-dependent fractional bleach losses X for low (red) and high (blue) push energies (Suppl. Fig. 9c). We fit the fractional bleach loss to $X(a, J) = 1 - \exp(-aJ)$ to account for saturation of the bleach loss and find the best-fit parameters for low $\hat{a}_{\mathrm{low}}$= 0.06 $\mathrm{cm^2mJ^{-1}}$ and high $\hat{a}_{\mathrm{high}}$= 0.11 $\mathrm{cm^2mJ^{-1}}$ push energies, respectively. Using these best-fit parameters, we fit the 2 sets of apparent trapping probabilities in Suppl. Fig. 9b to $P'_{\mathrm{trap}} = P_{\mathrm{trap}} + bJ^n/X(\hat{a}, J)$, with $n_{\mathrm{low}} = 5$ and $n_{\mathrm{high}} = 3$, P_{trap}, and b as fit parameters (Suppl. Fig. 9b solid lines). There is a good match between data and model, and we conclude that indeed multiphoton absorption explains the increase of P'_{trap} at higher push fluences. By minimizing the push fluence, we can measure the real trapping probability $P'_{\mathrm{trap}} = P_{\mathrm{trap}}$ from a PPP measurement directly. As an additional control experiment, we independently measure the absorbance bleach in a push–probe experiment (Suppl. Fig. 9d; 0.95 eV push energy), where we observe a cubic dependence of the absorbance bleach with push fluence. Indeed, the apparent trapping probability P'_{trap} starts to increase (Suppl. Fig. 9b) from the low-fluence limit P_{trap}, when multiphoton absorption becomes significant (Suppl. Fig. 9d). The real trapping probabilities P_{trap} are 7.5% for a push photon energy of 0.52 eV and 10.5% for a push photon energy of 0.95 eV. The contribution of hot-carrier trapping to the apparent trapping probability is 95% (relative) for the data in Fig. 2a of the main text.

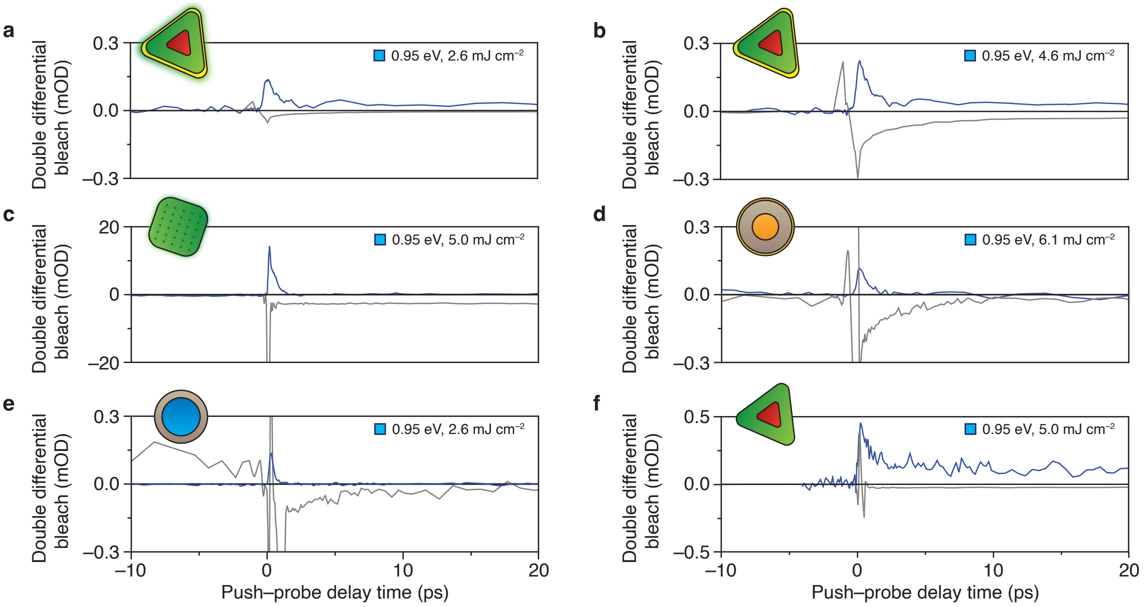

Supplementary Figure 10 | Push-induced optical artefacts. **(a)** Transient double-differential absorbance for a PPP experiment on InP/ZnSe/ZnS QDs (0.95 eV, 2.6 mJ cm^{-2}), which is the difference between the PPP absorbance bleach and the pump–probe (PP) absorbance bleach. We investigate the effect of the push without pump by measuring the push–probe trace at 7 mJ cm^{-2} (pump blocked). We reconstruct the push–probe trace at 2.6 mJ cm^{-2} (gray) using the cubic fluence dependence of 3-photon absorption to scale the data. The derivative-like feature at the arrival of the push probe is due to the optical Stark effect. For push–probe delays $t > 5$ we observe a small negative double-differential absorbance, which indicates multiphoton absorption induced by the push pulse. In a PPP experiment, we measure the differential absorbance, so the PPP signal does not directly contain push–probe effects, such as the optical Stark effect. **(b)** Same as **a**, but for a push fluence of 4.6 mJ cm^{-2}. The push–probe transient at 4.6 mJ cm^{-2} (gray) is again reconstructed from a 7 mJ cm^{-2} push–probe experiment. **(c)–(f)** Same as **a/b**, but for $CsPbBr_3$ **c**, CdSe/CdS/ZnS **d**, $CuInS_2$/CdS **e**, InP/ZnSe **f**. In all cases, the push-induced artefact is successfully subtracted by the differential measurement scheme.

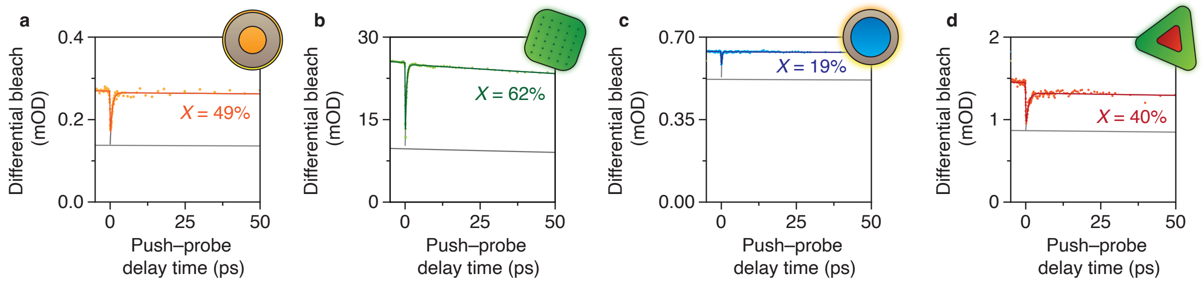

Supplementary Figure 11 | Non-normalised pump–push–probe experiments. **(a)** Non-normalised pump–push–probe experiment on CdSe/CdS/ZnS QDs, showing a fractional bleach recovery $X = 49\%$.**(b)–(d)** Same as **a**, but for **b** $CsPbBr_3$ perovskite nanocrystals ($X = 62\%$), **c** $CuInS_2$/CdS ($X = 19\%$), and **d** InP/ZnSe ($X = 40\%$). Bleach is averaged over the band-edge absorption band at 1.90–2.10 eV, 2.42–2.46 eV, 2.00–2.10 eV, and 2.12–2.15 eV for panels **a–d**, respectively.

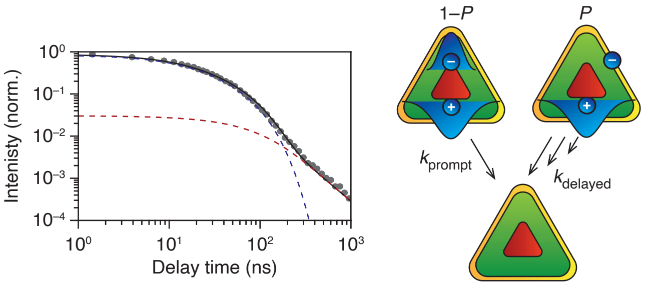

Supplementary Figure 12 | Characterizing the fraction of delayed emission. Transient-photoluminescence measurement of InP/ZnSe/ZnS QDs (left) at the emission maximum ($E_{\mathrm{det}} = 2.02$–2.05 eV) on a double-logarithmic scale. Most of the emission can be described by simple exponential decay dynamics (blue dashed line), attributed to prompt emission of delocalized excitations with decay constant k_{prompt}. Part of the emission is slow compared to prompt emission (red dashed line) and is attributed to free-to-bound recombination with "delayed" and distributed decay dynamics. To fit the total decay curve (Fig. 3), we first determine the prompt decay rates $k_{\mathrm{prompt},i}$ and corresponding amplitudes $A_{\mathrm{prompt},i}$ from a biexponential fit between delay times $t \in [0, 150]$ ns. Next, we determine the power-law slope α between delay times $t \in [300, 1000]$ ns. Finally, we fit the fraction P of delayed emission from fitting the transient-PL measurement to

$$I(t) \propto (1-P)\left(A_{\mathrm{prompt},1}\mathrm{e}^{-k_{\mathrm{prompt},1}t} + A_{\mathrm{prompt},2}\mathrm{e}^{-k_{\mathrm{prompt},2}t}\right) + P(\alpha-1)\left(\frac{\overline{k}_{\mathrm{prompt}}t}{2}\right)^{1-\alpha}\left[\Gamma(\alpha) - \Gamma\left(\alpha, \frac{\overline{k}_{\mathrm{prompt}}t}{2}\right)\right]t^{-\alpha}, \tag{37}$$

where $\overline{k}_{\mathrm{prompt}}$ is the amplitude-weighted average prompt decay rate, and $\overline{k}_{\mathrm{prompt}}/2$ is the assumed upper limit for the free-to-bound recombination rate in the power-law distribution of delayed recombination rates k_{delayed}[S10]. In Fig. 3d in the main text, we use the fitting procedure described above to find P at varying detection energies E_{det}. The delayed-emission spectrum is given by $I_{\mathrm{del}}(E_{\mathrm{det}}) = P(E_{\mathrm{det}})I_{\mathrm{tot}}(E_{\mathrm{det}})$, where I_{tot} is the total emission spectrum presented in Fig. 1b of the main text.

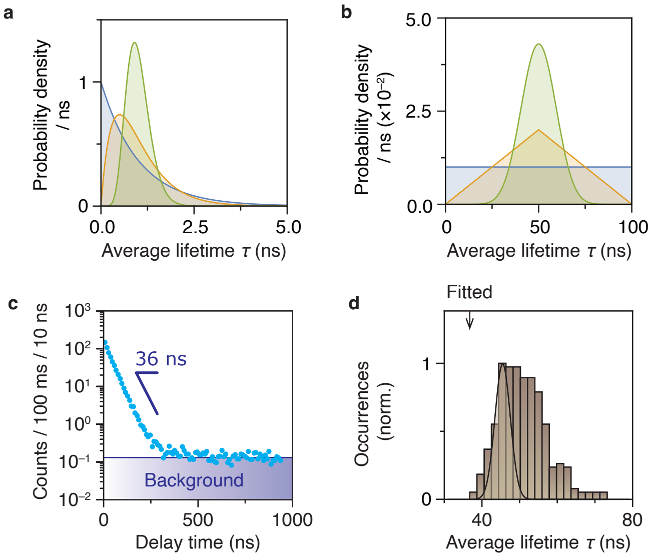

Supplementary Figure 13 | Contribution of flat background to the average lifetime. In our single-QD experiments, we monitor fluctuations of the excited-state lifetime (Fig. 4c) by subdividing the total experiment in time bins, for which we calculate the average delay time with the laser pulse. This is also commonly referred to as the average lifetime, because for single-exponential decay we would exactly retrieve the lifetime of the excited state. However, in an experiment, we detect relatively few (10^2–10^3) photons per time bin, and therefore we expect significant uncertainty in the determination of the average lifetime. Here, we investigate the uncertainty in the determination of the average lifetime and the influence of a flat background signal. **(a)** We first consider an exponential distribution with lifetime $\overline{\tau} = 1$ ns. If we sample this distribution once (using the timing of $n = 1$ photon), and repeat this many times, we retrieve the exponential distribution that is commonly plotted as the PL decay curve of an emitter (Suppl. Fig. 13a, blue). The "average lifetime" value estimated using information from multiple photons ($n > 1$), has a different probability distribution. For $n = 2$, we can find the probability distribution $P(\tau, 2)$ (Suppl. Fig. 13a, orange) by integrating over all possible delay times τ_1 and τ_2 of the two photons—drawn from the exponential distribution—that yield an average lifetime $\tau = (\tau_1 + \tau_2)/2$:

$$P_{\text{exp}}\Big(\tau = \frac{\tau_1 + \tau_2}{2}, 2\Big) = (\overline{\tau})^{-2} \int_0^{\tau} \mathrm{d}\tau_1 \mathrm{e}^{-\tau_1/\overline{\tau}} \mathrm{e}^{-(2\tau - \tau_1)/\overline{\tau}} = (\overline{\tau})^{-2} \tau \mathrm{e}^{-2\tau/\overline{\tau}}. \tag{38}$$

We can repeat this process to generate the probability distribution $P_{\text{exp}}(\tau, n)$ of finding an average lifetime τ using information from n photons (Suppl. Fig. 13a, green $n = 10$). For large n, the probability distribution approaches a normal distribution $P_{\text{exp}}(\tau, n) = \mathcal{N}(\overline{\tau}, \overline{\tau}^2/n)$, in agreement with the central limit theorem. The width of the normal distribution, *i.e.* the error in measuring the lifetime, decreases with increasing n. **(b)** In an experiment, stray light enters our optical setup which contributes to a flat background. To find the "average lifetime" of stray-light photons only, we can repeat the procedure introduced in equation 38 for a uniform distribution between $t \in [0, T]$ ns, where T is the maximum delay time included in the average-lifetime procedure. Suppl. Fig. 13b shows the result for $n = 1$ (blue), $n = 2$ (orange), and $n = 10$ flat background photons, and $T = 100$ ns. Also here, the probability distribution $P_{\text{flat}}(\tau, T, n)$ approaches a normal distribution $P_{\text{flat}}(\tau, T, n) = \mathcal{N}\Big(\frac{T}{2}, \frac{T^2}{12n}\Big)$ for large n. **(c)** Suppl. Fig. 13c shows the decay curve of the single QD presented in the main text (Fig. 3), constructed from all 100-ms bin with average lifetimes $\tau \in [40, 50]$ ns. The total number of photons per 100 ms is $n_{\text{tot}} = 632$ on average and we fit an excited-state lifetime of $\overline{\tau} = 36$ ns. We extract the contribution of flat background $n_{\text{flat}} = 13$ and QD signal $n_{\text{exp}} = 619$ on average. **(d)** Using the contributions of a flat background $n_{\text{flat}} = 13$ and QD signal $n_{\text{exp}} = 619$, we compute the probability distribution of the average lifetime. We convolve the approximately normal distributions for P_{flat} ($T = 1$ μs) and P_{exp} to arrive at

$$P_{\text{tot}}(\overline{\tau}, T, n_{\text{flat}}, n_{\text{exp}}) = \mathcal{N}\Big(\frac{n_{\text{flat}}\mu_{\text{flat}} + n_{\text{exp}}\mu_{\text{exp}}}{n_{\text{flat}} + n_{\text{exp}}}, \frac{n_{\text{flat}}^2\sigma_{\text{flat}}^2 + n_{\text{exp}}^2\sigma_{\text{exp}}^2}{(n_{\text{flat}} + n_{\text{exp}})^2}\Big), \tag{39}$$

where $\mu_{\text{flat}} = T/2$ and $\mu_{\text{exp}} = \overline{\tau}$. This probability distribution is a normal distribution with n-weighted mean and n^2-weighted variance. Suppl. Fig. 13d shows the histogram of average lifetimes of the single QD and the computed probability distribution (equation 39, black line). We observe that the flat background shifts the peak average lifetime to larger values compared to the fitted lifetime. Moreover, the experimental histogram of average

lifetimes is much broader than our computed probability distribution. This shows that real fluctuations of the excited-state lifetime occur in the experiment, *i.e.* $\overline{\tau}$ is not constant.

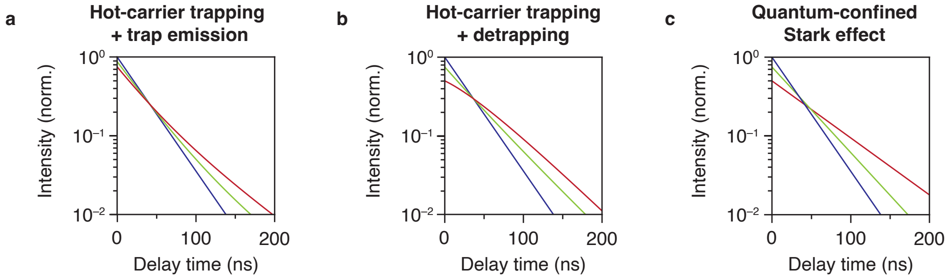

Supplementary Figure 14 | Trap emission, charge-carrier detrapping, and the Stark effect. (a) Schematic transient-PL measurements of a single QD with varying hot-carrier trapping probabilities $P_{\text{trap}} = 0\%$ (red), 25% (green), and 50% (blue), a radiative rate of $k_{\text{r}} = 1/30$ ns), and a free-to-bound radiative rate of $k_{\text{trap}} = 1/60$ ns. **(b)** Same as **a**, but here the trap state itself is optically inactive. Instead, delayed emission originates release of the trapped carrier to the band-edge state with a rate constant $k_{\text{rel}} = 1/(45$ ns) which results in band-edge emission. Consequently, we observe a significant rise in the transient-PL measurement at high trapping probabilities (blue) due to an ingrowth of the band-edge population on the timescale of charge-carrier release. **(c)** Schematic transient-PL measurements of a single QD with varying radiative decay rates of $k_{\text{r}} = 1/(30$ ns) (red), 1/(45 ns) (green), and 1/(60 ns) (blue) due to the quantum-confined Stark effect. The decay dynamics presented in panels **b,c** are inconsistent with the single-QD measurements in Fig. 4, which show biexponential decay dynamics similar to panel **a**.

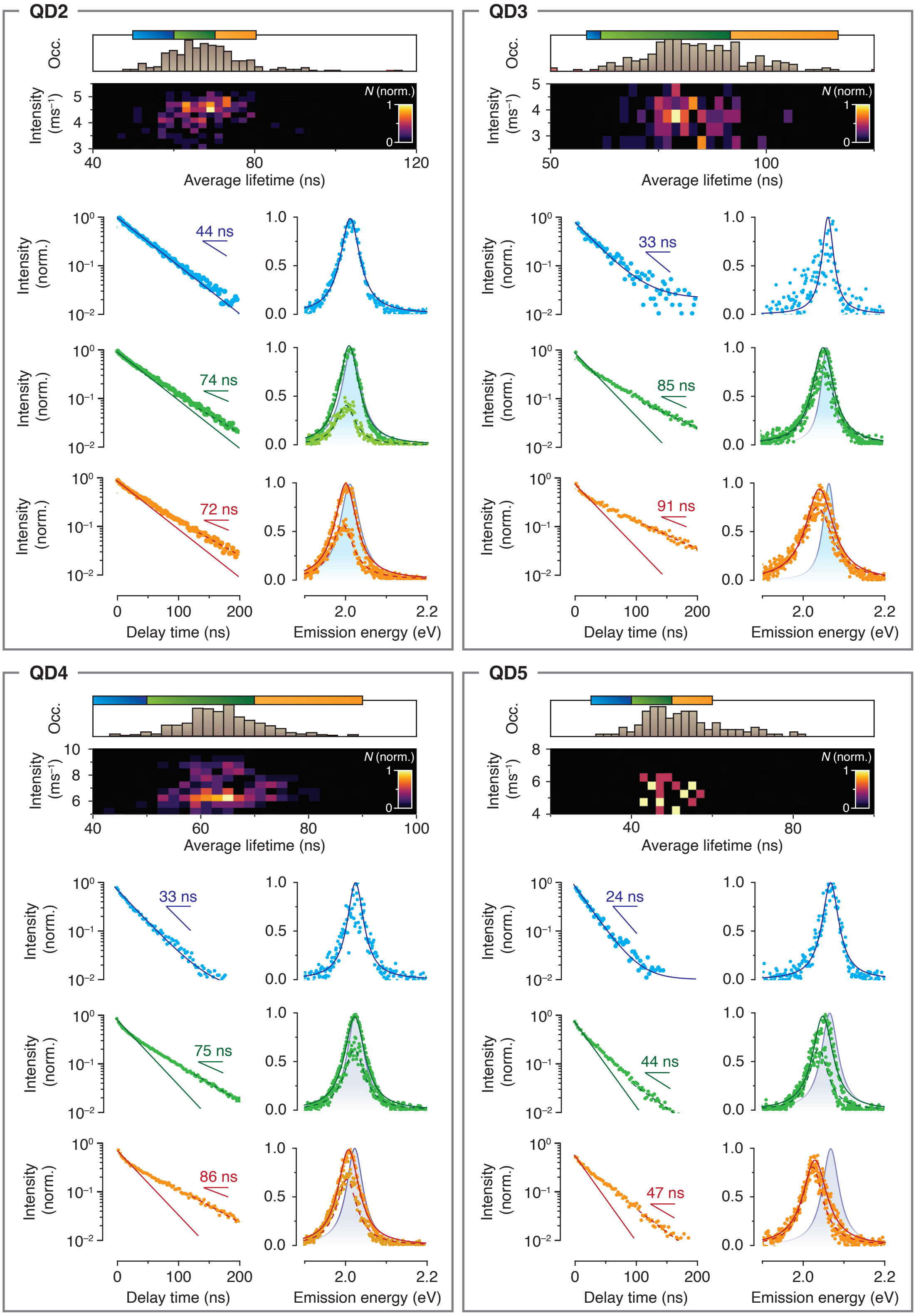

Supplementary Figure 15 | Single QDs 2–5. (Top) Lifetime–intensity distribution and lifetime distribution, indicating moments of fast (blue), intermediate (green), and long (orange) lifetimes. **(bottom)** Decay curves (left) and emission spectra (right) of moments of short (blue), intermediate (green), and long (orange) lifetime.

Supplementary Note 3 – Lifetime fluctuations in different semiconductor nanocrystals

We observed no hot-carrier losses from the pump–push–probe experiments in the main text (Figs. 2c–e) for $CsPbBr_3$ perovskite, CdSe/CdS/ZnS, and $CuInS_2$-based materials. In Fig. 4 of the main text, we show signatures of fluctuating hot-carrier trapping for single InP/ZnSe/ZnS QDs. Earlier reports also have shown fluctuating excited-state lifetimes for different materials. Here, we introduce measurements on single $CsPbBr_3$ perovskite nanoplatelets (NPLs) and re-analyze measurements from previous reports from our group[S11,S12], to show that different mechanisms are responsible for the fluctuating lifetimes.

3.1 $CsPbBr_3$ perovskite nanoplatelets

Suppl. Fig. 16a shows decay curves of selected moments of a short (blue) and long average lifetime (red) for a single 6-monolayer $CsPbrBr_3$ nanoplatelet. During short-lifetime moments, we observe single-exponential decay with an excited-state lifetime τ = 4.7 ns (solid blue line). During moments of long lifetimes, the decay curve follows biexponential decay with lifetime components of 2.0 ns and 30 ns. Interestingly, the short-lifetime component is significantly shorter (2.0 ns) than during moments of short lifetime (4.7 ns). This indicates opening and closing of a trapping pathway from the band edge, which competes with radiative recombination k_r, increasing the prompt decay rate to $k_{prompt} = k_r + k_{trap}$ (Suppl. Fig. 16b). Trapping from the band edge results in slow emission because of the slow release (or direct recombination) of the localized carrier. This mechanism is qualitatively different than observed in the InP/ZnSe/ZnS QDs since trapping competes with radiative recombination, not cooling.

3.2 CdSe/CdS core–shell QDs

Suppl. Fig. 16c shows decay curves of selected moments of a short (blue) and long average lifetime (red) for a single CdSe/CdS core–shell QD re-analyzed from Ref. S10. Both decay curves follow a single-exponential decay with lifetimes of 39 ns (blue) and 66 ns (red). The corresponding emission spectrum during moments of slow decay (Suppl. Fig. 16d, red) is redshifted by 17 meV and broader (FWHM = 85, compared to FWHM = 59 meV for the blue emission spectrum.) These observations are consistent with the quantum-confined Stark effect, where an electric field polarizes the exciton, which retards and redshifts the emission (Suppl. Fig. 16e). This mechanism is qualitatively different than observed in our InP/ZnSe/ZnS QDs, since the quantum-confined Stark introduces slower but *single-exponential* decay dynamics.

3.3 $CuInS_2$/CdS core–shell QDs

Suppl. Fig. 16f shows decay curves of selected moments of a short (blue) and long average lifetime (red) for a single $CuInS_2$/CdS (CIS/CdS) core–shell QD re-analyzed from Ref. S11. Both decay curves follow single-exponential decay with lifetimes of 342 ns (blue) and 455 ns (red). Both fitted lifetimes are considerably longer compared to the other semiconductor materials studied in this work. Emission from CIS originates from a charge-separated excited state (Suppl. Fig. 16h). The difference with the InP/ZnSe/ZnS QDs is that for these QDs the trapping probability equals unity $P_{trap} = 1$, and no band-edge emission is observed. This free-to-bound recombination mechanism results in a significantly broader emission spectrum both when the lifetime is short (FWHM = 195 meV) and long (FWHM = 216 meV), due to boosted phonon-coupling of localized charge carriers. The negative correlation between lifetime and emission energy is consistent with the quantum-confined Stark effect.

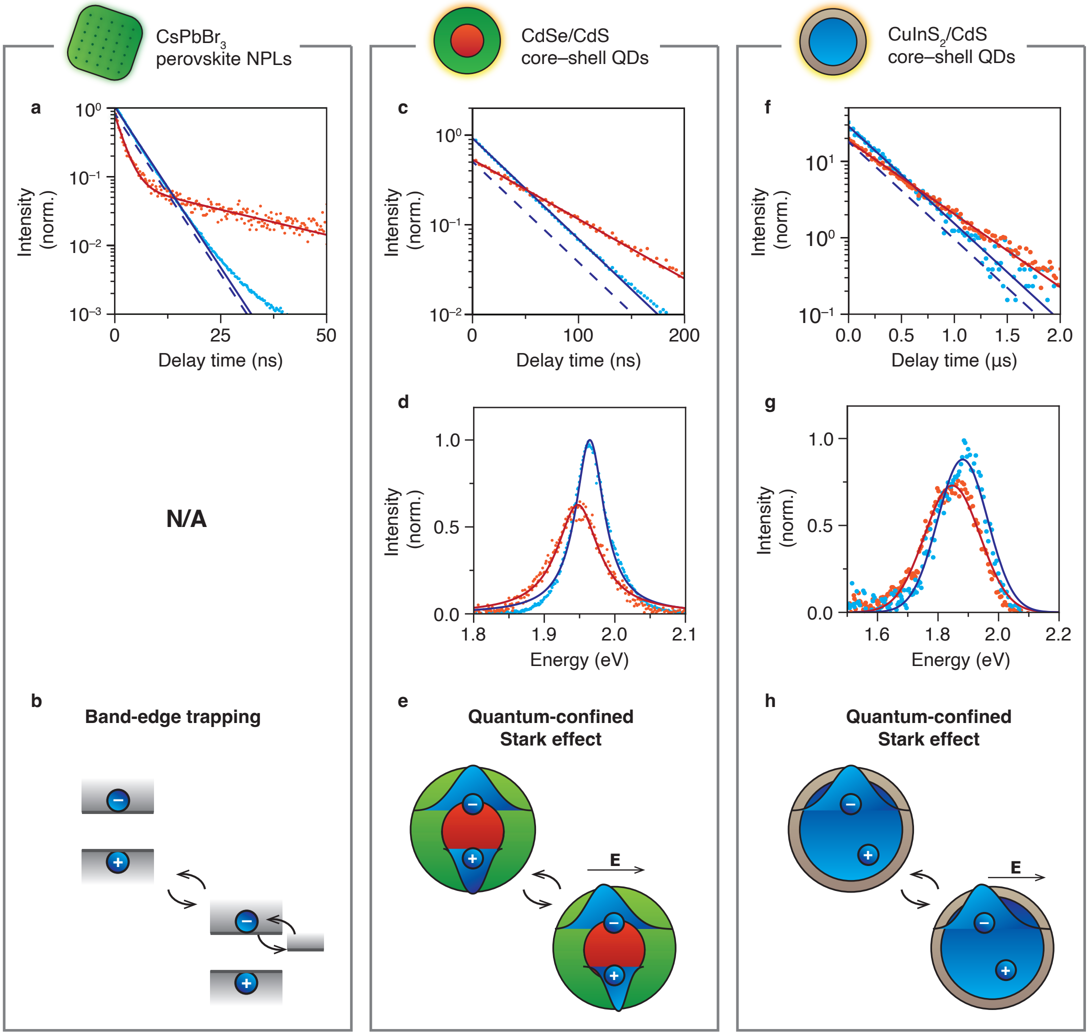

Supplementary Figure 16 | Single-QD measurements of different materials. (a–b) $CsPbBr_3$ perovskite nanoplatelets. **(c–e)** CdSe/CdS core–shell QDs, reproduced from Ref. S11. **(f–h)** $CuInS_2$/CdS core–shell QDs, reproduced from Ref. S12.

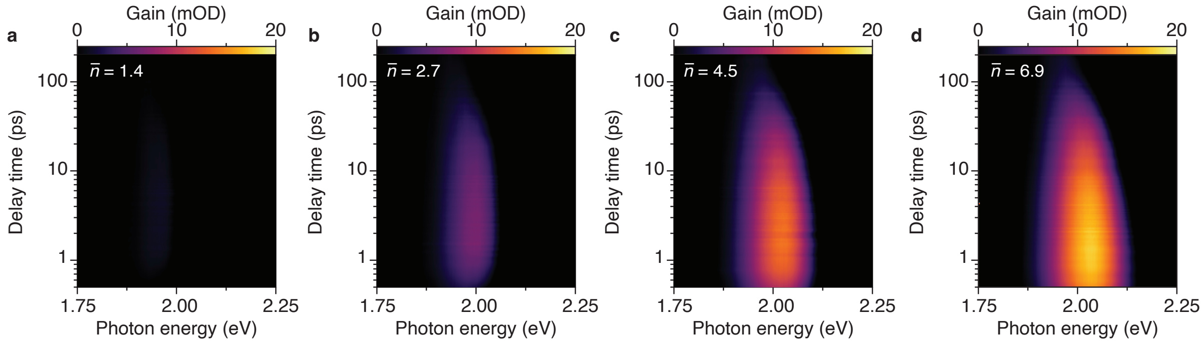

Supplementary Figure 17 | Time-dependent gain spectra of InP/ZnSe/ZnS QDs. **(a)** Time-dependent gain (masked excited-state absorption $A < 0$) spectra at $\overline{n} = 1.4$. **(b)–(d)** Same as a, but for $\overline{n} = 2.7$, $\overline{n} = 4.5$, and $\overline{n} = 7.1$. We observe that the gain blueshifts and increases in magnitude as a function of $\overline{n}$, but for each experiment redshifts as a function of time after excitation.

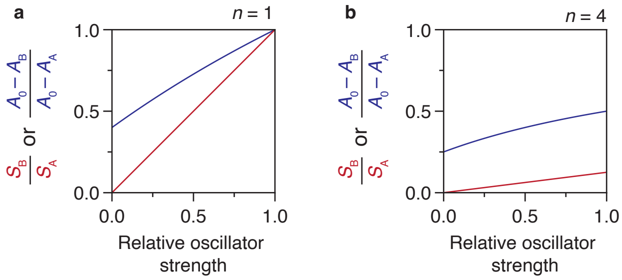

Supplementary Figure 18 | Influence of hot-carrier trapping on absorption and stimulated emission. **(a)** Relative absorption loss $(A_0 - A_\mathrm{B})/(A_0 - A_\mathrm{A})$ (blue) and stimulated emission strength $S_\mathrm{B}/S_\mathrm{A}$ (red) as a function of the relative oscillator strength x of the free-to-bound transition for scenarios A (no trapping) compared B (trapping) for exactly 1 excitation $n = 1$. **(b)** Same as **a**, but for $n = 4$. In both cases, the relative loss, due to hot-carrier trapping, of stimulated emission is larger compared to the loss of absorption bleach. This shows that stimulated emission is more sensitive to hot-carrier trapping compared to absorption. This in turn explains why the Stokes-shifted gain in InP-based QDs, which relies mostly on stimulated emission and not on absorbance bleach, saturates so easily.

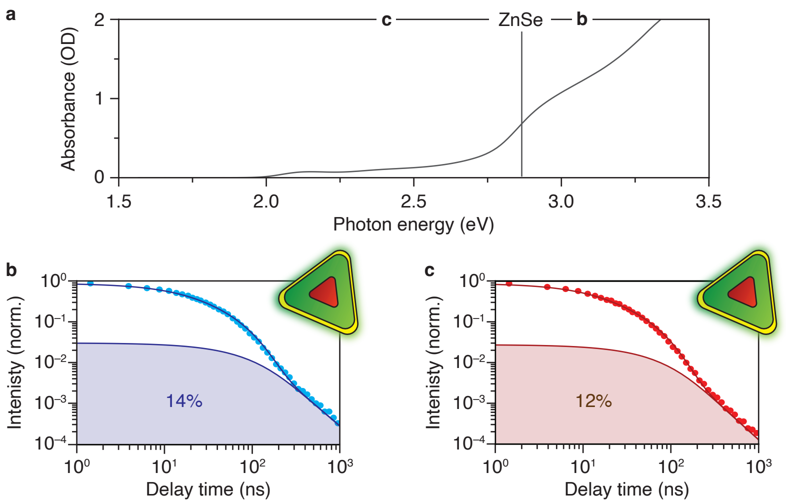

Supplementary Figure 19 | Dependence of delayed emission on InP core vs. ZnSe shell excitation. **(a)** Absorbance spectrum of the InP/ZnSe/ZnS QD sample. For photon energies $E > 2.84$ eV, the absorbance increases because of additional absorption by the ZnSe shell material. Below that photon energy, we mainly have absorption by the InP core. **(b)** Transient-PL measurement of the InP/ZnSe/ZnS QDs upon excitation into the ZnSe shell, at an energy of $E_{\text{laser}} = 3.1$ eV. **(c)** Same as **b**, but for 515-nm excitation ($E_{\text{laser}} = 2.4$ eV).

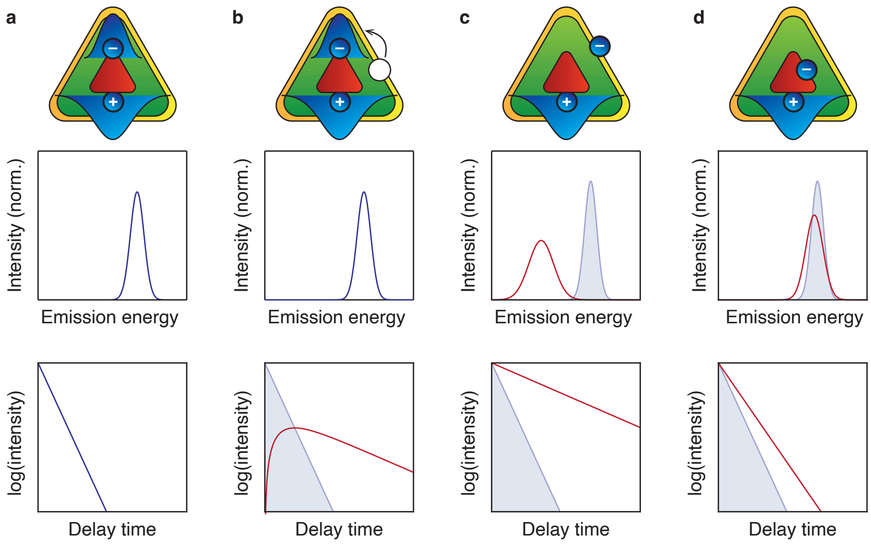

Supplementary Figure 20 | Possible mechanisms of trap-related delayed emission. **(a)** A delocalized excitation in InP-based QDs gives rise to band-edge emission (middle) and decay (bottom). **(b)** Charge-carrier detrapping following by recombination of delocalised carriers yields the same emission spectrum as **a**. The decay dynamics (red) change as the emitting population is first fed by detrapping after which electron–hole recombination leads to PL. **(c)** Trap emission from a delocalised hole and an electron trapped on the QD surface. Because of the large reorganization energy and large static dipole moment, the emission spectrum is significantly redshifted and broadened compared to regular emission from a delocalised excitation. **(d)** Trap emission from a delocalised hole and a trapped electron on the core–shell interface. Here, we expect smaller reorganization energies and less phonon broadening. This combination of signatures is consistent with all our experimental results.

Supplementary references

(S1) Nguyen, A.T., La Plante, I.J., Ippen, C., Ma, R. & Kelley, D.F. Extremely slow trap-mediated hole relaxation in room-temperature InP/ZnSe/ZnS quantum dots. *J. Phys. Chem. C* **125**, 4110–4118 (2021).

(S2) Sousa Velosa, F., Van Avermeat, H., Schiettecatte, P., Mingabudinova, L., Geiregat, P. & Hens, Z. State filling and stimulated emission by colloidal InP/ZnSe core/shell quantum dots. *Adv. Opt. Mater.* **10**, 2200328 (2022).

(S3) Hens, Z. & Moreels, I. Light absorption by colloidal semiconductor quantum dots. *J. Mater. Chem.* **22**, 10406 (2012).

(S4) Aspnes, D.E. & Studna, A.A. Dielectric functions and optical parameters of Si, Ge, GaP, GaAs, GaSb, InP, InAs, and InSb from 1.5 to 6.0 eV *Phys. Rev. B* **27**, 985–1009 (1983).

(S5) Adachi, S. & Taguchi, T. Optical properties of ZnSe. *Phys. Rev. B* **43**, 9569–9577 (1991).

(S6) Kozma, I.Z. & Krok, P. Direct measurement of the group-velocity mismatch and derivation of the refractive-index dispersion for a variety of solvents in the ultraviolet. *J. Opt. Soc. Am. B* **22**, 1479–1485 (2005).

(S7) Respekta, D., Schiettecatte, P., Giordano, N., De Vlamynck, N., Geiregat, P., Climente, J.I. & Hens, Z. Energy-level structure and band alignment in InP/ZnSe core/shell quantum dots. *ACS Nano*, in press, doi.org/10.1021/acsnano.5c02258 (2025).

(S8) Hendry, E., Koeberg, M., Wang, F., Zhang, H., de Mello Donega, C., Vanmaekelbergh, D. & Bonn, M. Direct observation of electron-to-hole energy transfer in CdSe quantum dots. *Phys. Rev. Lett.* **96**, 057408 (2006).

(S9) Prins, P.T., Spruijt, D.A.W., Mangnus, M.J.J., Rabouw, F.T., Vanmaekelbergh, D., de Mello Donega, C. & Geiregat, P. Slow hole localization and fast electron cooling in Cu-doped InP/ZnSe quantum dots. *J. Phys. Chem. Lett.* **13**, 9950–9956 (2022).

(S10) Rabouw, F.T., Kamp, M., van Dijk–Moes, R.J.A., Gamelin, D.R., Koenderink, A.F., Meijerink, A. & Vanmaekelbergh, D. Delayed exciton emission and its relation to blinking in CdSe quantum dots. *Nano Lett.* **11**, 7718–7725 (2015).

(S11) Hinterding, S.O.M., Vonk, S.J.W., van Harten, E.V.H. & Rabouw, F.T. Dynamics of intermittent delayed emission in single CdSe/CdS quantum dots. *J. Phys. Chem. Lett.* **12**, 4755–4761 (2020).

(S12) Hinterding, S.O.M., Mangnus, M.J.J., Prins, P.T., Jöbsis, H.J., Busatto, S., Vanmaekelbergh, D., de Mello Donega, C. & Rabouw, F.T. Unusual spectral diffusion of single $CuInS_2$ quantum dots sheds light on the mechanism of radiative decay. *Nano Lett.* **21** 658–665 (2021).
